# Suppression of exosomal miR-199a-3p, a differentially expressed miRNA in steroid-induced osteonecrosis of the femoral head, promotes cell proliferation, osteogenesis, and angiogenesis while inhibiting dexamethasone-induced apoptosis

**DOI:** 10.3389/fbioe.2026.1709739

**Published:** 2026-02-05

**Authors:** Tixiong Xia, Chengbin Yang, Xi Li, Tong Chen, Yingxing Xu

**Affiliations:** 1 Department of Orthopaedics, First Affiliated Hospital of Kunming Medical University, Kunming, Yunnan, China; 2 Research Center for Clinical Medicine, First Affiliated Hospital of Kunming Medical University, Kunming, China

**Keywords:** angiogenesis, bone marrow mesenchymal stem cells, differential expression profiles of RNAs, exosomes, osteogenesis

## Abstract

**Introduction:**

Steroid-induced osteonecrosis of the femoral head (SONFH) is a progressive and debilitating disorder caused by excessive glucocorticoid exposure. Dysfunction of bone marrow mesenchymal stem cells (BMSCs) and their exosome (Exos)-mediated signal transduction plays a key role in SONFH; however, the exact pathways involved remain under active investigation.

**Methods:**

The differential expression profiles of long non-coding RNAs (lncRNAs), microRNAs (miRNAs), and messenger RNAs (mRNAs) related to the Exos-mediated pathway in Exos derived from human BMSCs (hBMSCs) of patients with SONFH and control patients were analyzed by next-generation sequencing (NGS). The miR‐199a‐3p was identified as a differentially expressed miRNA, and its expression in BMSCs and their corresponding Exos was subsequently validated using quantitative real-time polymerase chain reaction. A series of functional experiments then confirmed that miR-199a-3p modulated osteoblasts (OBs) and human umbilical vein endothelial cells (HUVECs) activities via the Exos-mediated pathway, including cell proliferation, apoptosis, osteogenesis, and angiogenesis, with or without exposure to high-dose dexamethasone (Dex).

**Results:**

NGS results revealed a total of 6,953 differentially expressed ncRNAs, 260 differentially expressed miRNAs, 13,577 differentially expressed mRNAs were identified in hBMSCs from patients with SONFH compared to controls. In hBMSCs-Exos, 207 differentially expressed ncRNAs, 183 differentially expressed miRNAs, and 1,075 differentially expressed mRNAs were detected. Integrated analysis of transcripts expressed in both hBMSCs and hBMSCs-Exos identified 659 differentially expressed ncRNAs, 11 differentially expressed miRNAs, and 1,600 differentially expressed mRNAs. The results of bioinformatics analysis showed that these differentially expressed RNAs were involved in regulation of endocytosis, receptor-mediated endocytosis, response to extracellular stimuli, and bone mineralization. Furthermore, the validation results demonstrated that the suppression of miR-199a-3p promoted proliferation, osteogenesis, and angiogenesis, while inhibiting apoptosis of OBs and HUVECs activities and exposed to high-dose Dex.

**Discussion:**

This study identified differential expression profiles of ncRNAs, miRNAs and mRNAs related to the Exos-mediated pathway in SONFH through integrated analysis, and further demonstrated the negative role of miR‐199a‐3p in SONFH, involving proliferation, osteogenesis, and angiogenesis, as well as the regulation of apoptosis of OBs and HUVECs exposed to high-dose Dex through the hBMSCs-Exos-mediated pathway. Therefore, targeting miR‐199a‐3p may enhance the therapeutic efficacy of Exos-based treatments for SONFH.

## Introduction

Steroid-induced osteonecrosis of the femoral head (SONFH) is a pathological condition characterized by ischemia and subsequent necrosis of bone tissue in the femoral head, typically triggered by prolonged or high-dose exposure to glucocorticoids (Motta et al., 2022). Glucocorticoids impair the normal functions of osteocytes and vascular endothelial cells through multiple mechanisms, resulting in reduced bone perfusion, increased osteocyte apoptosis, and degradation of the trabecular bone structure (Kerachian et al., 2009; Gado et al., 2022). These pathological changes ultimately lead to collapse of the femoral head and progressive loss of joint function. The underlying pathogenesis is primarily attributed to vascular injury, impaired microcirculation, and the ensuing apoptosis and necrosis of osteogenic cells.

Exosomes (Exos) are a class of extracellular vesicles ranging from approximately 30–150 nm in diameter, generated and secreted by various cell types, including bone marrow mesenchymal stem cells (BMSCs), immunocytes, and epithelial cells. These vesicles are enriched with diverse biomolecules, including proteins, lipids, and nucleic acids such as non-coding RNAs (ncRNAs), microRNAs (miRNAs), and messenger RNAs (mRNAs), and serve as key mediators of intercellular communication by modulating the function and behavior of recipient cells (Pant et al., 2012; Urbanelli et al., 2013; Gurung et al., 2021). Previous studies have shown that Exos derived from BMSCs are involved in the therapeutic mechanisms of SONFH. As natural nanocarriers, Exos contribute to the treatment of SONFH by delivering functional biomolecules to target cells (Kim et al., 2017; Huang et al., 2023). Additionally, Exos have been shown to regulate bone mineralization processes (Kim et al., 2017; Fang et al., 2019; Masaoutis and Theocharis, 2019). However, the precise mechanisms by which Exos influence osteogenesis, angiogenesis, cell proliferation, and apoptosis in target cells remain largely unclear. Notably, Exos mediate intercellular communication primarily by transporting genetic cargoes, thereby transmitting specific molecular information from donor cells to recipient cells via the extracellular space. Upon uptake, these cargoes modulate the functional and phenotypic characteristics of recipient cells. This process involves several key steps, including surface receptor interactions, membrane fusion, and receptor-mediated endocytosis in target cells (Gonda et al., 2019; Chen et al., 2021). Therefore, elucidating the specific genetic materials encapsulated and delivered by Exos is critical for understanding their mechanistic roles in cellular regulation, a process referred to as the “Exos-mediated pathway”.

As a result, next-generation sequencing (NGS) was employed in this study to identify differential gene expression profiles of ncRNAs, miRNAs, and mRNAs related to the Exos-mediated pathway in SONFH. This was achieved by integrating the differentially expressed genes from human BMSCs (hBMSCs) and their derived Exos (hBMSCs-Exos) obtained from patients with SONFH and controls. Among these, a significantly differentially expressed miRNA, miR-199a-3p, was identified. Its potential biological functions were predicted through bioinformatics analyses, including Gene Ontology (GO), Kyoto Encyclopedia of Genes and Genomes (KEGG) pathway analysis, and competing endogenous RNA (ceRNA) interaction network analysis. Finally, the role of miR-199a-3p-modified hBMSCs-Exo in regulating cell proliferation, apoptosis, osteogenesis, and angiogenesis was experimentally validated in this study.

## Materials and methods

### Isolation and culture of hBMSCs

This study was approved by the Ethics Committee of the First Affiliated Hospital of Kunming Medical University (Kunming, China). Bone marrow specimens were collected from three female patients diagnosed with SONFH (aged 45, 47, and 52 years) and two female patients with femoral neck fractures (aged 60 and 63 years), all of whom underwent total hip arthroplasty at the Department of Orthopedics, First Affiliated Hospital of Kunming Medical University. Written informed consent was obtained from all donors. Bone marrow-derived cells were isolated and purified using density gradient centrifugation, as previously described (Insausti et al., 2012). The cells were then cultured in Dulbecco’s Modified Eagle’s Medium (DMEM; Solarbio, Beijing, China) supplemented with 10% (v/v) fetal bovine serum (FBS; Gibco, Thermo Scientific, Australia) and 100 U/mL penicillin-streptomycin (Solarbio, Beijing, China), under standard conditions at 37 °C in a humidified atmosphere containing 5% CO_2_. When the cultures reached approximately 90% confluency, the cells were passaged at a 1:2 ratio. All subsequent experiments were conducted using third-passage (P3) cells.

### Characterization of hBMSCs

Flow cytometry was used to evaluate the expression of surface markers on human hBMSCs using an ACEA NovoCyte 2060R flow cytometer (ACEA Biosciences, USA). Briefly, cells were enzymatically detached with trypsin, collected by centrifugation, and resuspended in cold phosphate-buffered saline (PBS) containing 1% fetal bovine serum (FBS). The cell suspension was adjusted to a concentration of 1 × 10^6^ cells/mL and incubated with the following fluorochrome-conjugated antibodies for 30 min at 37 °C in the dark: anti-CD34-FITC (5 µL), anti-CD73-FITC (5 µL) (Elabscience, China), anti-CD45-FITC (5 µL), and anti-CD90-FITC (5 µL) (Proteintech Group, China). After incubation, cells were washed three times with cold PBS, and 100 µL of the stained single-cell suspension was subjected to flow cytometric analysis.

### Isolation and characterization of Exos

After 48 h of culture in serum-free mesenchymal stem cell medium, the conditioned media from both cell groups were collected for Exos isolation. Exos were extracted and purified using differential ultracentrifugation according to established protocols (Thery et al., 2006; Li et al., 2017). The resulting exosomal pellets were resuspended in PBS and either used immediately or stored at −80 °C for subsequent analyses.

Exos characterization was performed using multiple techniques: (1) nanoparticle tracking analysis (NTA) was used to determine particle size distribution and concentration (Gurunathan et al., 2019); (2) transmission electron microscopy (TEM) was used to observe the ultrastructural morphology. In the TEM analysis, 10 µL of isolated Exos were applied onto a copper mesh grid for 5 min, and excess liquid was removed using filter paper. After blotting and air-drying, the samples were stained with 10 µL of 2% uranyl acetate and examined using an HT7700 transmission electron microscope (Hitachi, Japan); (3) Western blot analysis was performed to detect exosomal marker proteins, including TSG101 and CD63 (Lässer et al., 2012).

### NGS analysis

Total RNA was extracted from hBMSCs and their corresponding Exos in both groups using TRIzol™ reagent (Invitrogen, USA), following the manufacturer’s protocol. RNA concentration and purity were measured using a NanoDrop 2000 spectrophotometer (Thermo Fisher Scientific, USA), while RNA integrity was evaluated using an Agilent 2100 Bioanalyzer (Agilent Technologies, USA). Only samples with high RNA integrity, defined as an RNA Integrity Number (RIN) greater than 7.0, were selected for subsequent analyses.

Total RNA was fragmented and reverse-transcribed into complementary DNA (cDNA), followed by end-repair, adapter ligation, and PCR amplification to enrich the sequencing libraries. The final libraries were quantified, normalized, and subjected to high-throughput paired-end sequencing using the Illumina HiSeq™ 2500 platform (Illumina, USA).

Raw sequencing reads were preprocessed using Trimmomatic (v0.39) to remove low-quality bases and adapter sequences, generating high-quality clean reads. These clean reads were then aligned to the GRCh38 human reference genome using HISAT2 (v2.2.1). Transcript assembly and quantification were performed with StringTie (v2.1.4). Differential expression analysis between groups was conducted using the DESeq2 package (v1.30.0) in R, applying thresholds oflog_2_ (fold change) > 1 and adjusted *P*-value <0.05. The experiment was conducted and data were analyzed in triplicate.

The comprehensive differential gene expression profiles associated with the Exos-mediated pathway were obtained by identifying differentially expressed transcripts present in both hBMSCs and their derived Exos.

### Bioinformatics analysis

GO and pathway enrichment analyses of the differentially expressed genes were performed using DAVID Bioinformatics Resources 6.8 (https://david.ncifcrf.gov/). GO analysis was conducted to clarify the functional roles of the differentially expressed genes between the two groups, covering three categories: Biological Process (BP), Cellular Component (CC), and Molecular Function (MF). Pathway enrichment analysis was carried out based on the KEGG database.

### Prediction of miR-199a-3p-targeted lncRNAs and mRNAs

Using integrated bioinformatics analyses, potential long lncRNAs and mRNAs targeted by miR-199a-3p were predicted via the ENCORI database (http://starbase.sysu.edu.cn/index.php). Candidate targets were then further refined, and their functions analyzed through a comprehensive review of the NCBI database (https://www.ncbi.nlm.nih.gov/), focusing on roles related to osteogenesis, angiogenesis, cell proliferation, apoptosis, as well as Exos biogenesis and secretion.

### Validation of miR-199a-3p in hBMSCs and hBMSCs-Exo by qRT-PCR analysis

Differentially expressed miR-199a-3p was selected to validate the NGS results by qRT-PCR. Total RNA was extracted from hBMSCs and their corresponding Exos from both groups using a TransZol Up (TransGen, China). Subsequently, the extracted RNA was reverse-transcribed into cDNA, and qRT-PCR was performed using a SYBR Green Hairpin-it miRNA qRT-PCR Kit (GenePharma, China) according to the manufacturer’s instructions. RNA concentration was determined using a modified approach combining absorbance (Nanodrop spectrophotometer, 160 ± 10 ng/μL) (Thermo Fisher, USA) with fluorescence (the Qubit miRNA HS assay, 12 ng/μL) to ensure accurate determination of the amount of miR-199a-3p available for the reverse-transcription step. The qRT-PCR was performed on a LightCycler® 96 Instrument (Roche, Schweiz). The relative expression levels of miR-199a-3p (primers: forward 5′-AGC​TTC​TGG​AGA​TCC​TGC​TCC-3’; reverse 5′-TCC​CTT​GCC​CAG​TCT​AAC​CAA-3′) in cellular and exosomal samples were quantified by qRT-PCR. Relative gene expression levels of miR-199a-3p between groups were calculated using the comparative 2^−ΔΔCT^ method according to the previous report (Livak and Schmittgen, 2001), and normalized to U6 small nuclear RNA (Ribobio, China). Statistical analysis was performed using one-way ANOVA, and *P* < 0.05 was considered statistically significant. Values that reached statistical significance but did not meet the conventional 2^−ΔΔCT^ fold-change cut-offs (<0.5 for downregulation, >2 for upregulation) are reported as indicative of a trend rather than strict biological regulation. This approach ensures that differences are interpreted in the context of both statistical significance and biological relevance.

### Oligonucleotide transfection

miR-199a-3p expression in hBMSCs was modulated via oligonucleotide transfection using Cy3^+^-labeled mimics and inhibitors purchased from RiboBio (Guangzhou, China). The sequences were as follows: mimics sense strand (5′-ACA​GUA​GUC​UGC​ACA​UUG​GUU​A-3′); mimics antisense strand, 5′-ACC​AAU​GUG​CAG​ACU​ACU​GUU​U-3’; inhibitor (5′-mUmAmAmCmCmAmAmUmGmUmGmCmAmGmAmCmUmAmCmUmGmU-3′); mimics NC (mimics Negative Control) sense strand (5′-UUU​GUA​CUA​CAC​AAA​AGU​ACU​G-3′); mimics NC antisense strand (5′-GUA​CUU​UUG​UGU​AGU​ACA​AAU​U-3′); inhibitor NC (inhibitor Negative Control) (5′-mCmAmGmUmAmCmUmUmUmUmGmUmGmUmAmGmUmAmCmAmAmA)-3′). Transfection was performed using Lipofectamine 2000 (Invitrogen, USA) as the transfection enhancer. Transfection was performed in 6-well plates using serum- and antibiotic-free DMEM (Gibco, USA), with Lipofectamine 2000 added at 5 μL per well. The final concentrations were 50 nM for the mimics and mimics NC, and 100 nM for the inhibitor and inhibitor NC. According to the manufacturer’s instructions, the culture medium was replaced 6 h after transfection. The morphology of Cy3 positive cells was examined under a fluorescence microscope 24 h later, and transfection efficiency in hBMSCs was subsequently evaluated by qRT-PCR. Exos from the different treatment groups were then isolated following the previously described protocol.

### Experimental grouping

Human immortalized OBs (Cellverse, China) and human umbilical vein endothelial cells (HUVECs) (ScienCell, USA) were used to investigate the biological functions of miR-199a-3p, with or without exposure to high-dose dexamethasone (Dex) (10^–6^ mol/L for OBs; 10^–4^ mol/L for HUVECs) (Solarbio, China), including cell proliferation, apoptosis, osteogenesis, and angiogenesis. The specific treatments for each group were as follows:

1. Control: Untreated cells without any special treatment.
2. Mimics-Exo: Cells treated with Exos derived from cells transfected with miR-199a-3p mimics.
3. Inhibitor-Exo: Cells treated with Exos derived from cells transfected with miR-199a-3p inhibitors.
4. Exo: Cells treated with Exos derived from non-transfected (normal) cells.
5. miR-NCI-Exo: Cells treated with Exos derived from cells transfected with inhibitor negative control.
6. miR-NC-Exo: Cells treated with Exos derived from cells transfected with mimic negative control.
7. Dex: Cells treated with Dex only.
8. Dex + Mimics-Exo: Cells treated with both Dex and Exos derived from cells transfected with miR-199a-3p mimics.
9. Dex + Inhibitor-Exo: Cells treated with both Dex and Exos derived from cells transfected with miR-199a-3p inhibitors.
10. Dex + Exo: Cells treated with both Dex and Exos derived from normal cells.
11. Dex + miR-NCI-Exo: Cells treated with both Dex and Exos derived from cells transfected with inhibitor negative control.
12. Dex + miR-NC-Exo: Cells treated with both Dex and Exos derived from cells transfected with mimic negative control.

All experiments were conducted with three replicates per group and analyzed in triplicate.

### Crystal violet staining assay

Crystal violet staining was used to evaluate cell proliferation. Cells were seeded into 96-well plates at a density of 1 × 10^4^ cells per well and treated according to the experimental groupings described above. During the 7-day culture period, cells were stained daily with 0.1% crystal violet solution (Solarbio, China) in accordance with the manufacturer’s instructions. After staining, the dye was eluted with 33% pre-chilled acetic acid, and absorbance was measured quantitatively at 570 nm using a microplate reader (Allsheng, China).

### Flow cytometric analysis of apoptosis

Flow cytometric analysis was used to evaluate cell apoptosis. Cells were seeded into 6-well plates at a density of 1 × 10^6^ cells per well and treated according to the experimental groupings described above. Apoptosis was quantitatively assessed at four time points (days 1, 3, 5, and 7) using an Annexin-V/PI apoptosis detection kit (Elabscience, China), following the manufacturer’s instructions.

### TUNEL staining

TUNEL staining was performed to evaluate apoptosis morphologically. Cells were seeded into 6-well plates at a density of 1 × 10^6^ cells per well and treated according to the experimental groupings described above. At four time points (days 1, 3, 5, and 7), cells were stained using a TUNEL assay kit (Solarbio, China) following the manufacturer’s instructions. Apoptotic cells were observed and quantified by counting six randomly selected fields under a fluorescence microscope (Olympus, China). Quantification of TUNEL staining was performed using ImageJ (NIH, USA). Following background correction and threshold-based segmentation, nuclei were detected and counted with the “Analyze Particles” tool. DAPI-positive nuclei were used to determine the total cell number, whereas nuclei exhibiting colocalized TUNEL and DAPI signals were classified as apoptotic. The apoptotic index was expressed as the percentage of TUNEL-positive nuclei relative to the total number of DAPI-stained nuclei.

### Alkaline phosphatase (ALP) staining

ALP staining was used to evaluate the osteogenic differentiation capacity of OBs. Cells were seeded into 24-well plates at a density of 1 × 10^5^ cells per well and treated according to the experimental groupings described above for 5 days. Subsequently, ALP staining was conducted using a commercial ALP staining kit (Solarbio, China) following the manufacturer’s instructions to visualize cellular ALP activity.

### ALP activity assay

ALP activity assay was performed to quantitatively assess the osteogenic differentiation capacity of OBs. Cells were seeded into 24-well plates at a density of 1 × 10^5^ cells per well and treated according to the experimental groupings described above for 5 days. Subsequently, cellular ALP activity was measured using an ALP activity assay kit (Elabscience, China), following the manufacturer’s instructions.

### Alizarin Red S (ARS) staining

ARS staining was used to evaluate the osteogenic differentiation capacity of OBs. Cells were seeded into 24-well plates at a density of 1 × 10^5^ cells per well and treated according to the experimental groupings described above for 15 days. Subsequently, cells were stained with 0.1% ARS solution (Solarbio, China) and washed three times with PBS, following the manufacturer’s instructions. Mineralized matrix formation was then examined microscopically.

### Tube formation assay

The tube formation assay was used to evaluate the angiogenic capacity of HUVECs. Cells were seeded into 24-well plates pre-coated with Matrigel (NEST, China) at a density of 1 × 10^4^ cells per well, in accordance with the protocols of standard angiogenesis assay. After treatment according to the experimental groupings described above, cells were observed microscopically at 12 and 30 h to assess morphological and functional changes. Tube formation, including junction number and total tube length, was quantitatively analyzed using ImageJ software (NIH, USA).

### Statistical analysis

All statistical analyses were performed using SPSS version 19.0 (IBM, Armonk, NY, USA). One-way analysis of variance (ANOVA) was used to compare differences among three or more groups, while unpaired Student’s t-tests were applied for comparisons between two groups. In addition, the homogeneity test for variance was also performed. When the statistics exhibited heteroscedasticity, Tamhane’s T2 test was used. Data were expressed as mean ± standard deviation (mean ± SD). A *P*-value of less than 0.05 was considered statistically significant. All graphs were generated using GraphPad Prism version 10.0 (GraphPad Software, San Diego, CA, USA).

## Results

### Characterization of hBMSCs and Exos

**FIGURE 1:**
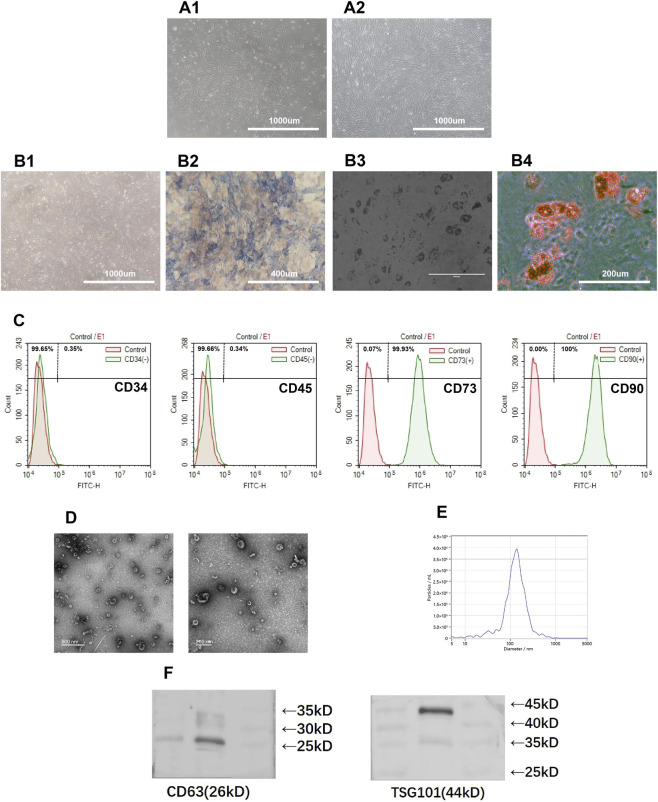
Characterization of hBMSCs and Exos. **(A)** Representative images showing morphology of hBMSCs at passage 3 under an inverted phase contrast microscope at 40% confluency **(A1)** and at 100% confluency **(A2)** (scale bar = 1,000 µm). **(B)** Osteogenic and adipogenic differentiation of hBMSCs: **(B1)** Morphology of hBMSCs induced by osteogenesis (scale bar = 1,000 µm); **(B2)** ALP staining (scale bar = 400 µm); **(B3)** Lipid droplets formation (scale bar = 400 µm); **(B4)** Oil red O staining (scale bar = 400 µm). **(C)** Phenotypic analysis of hBMSCs by flow cytometry (CD34, CD45, CD73, CD90). **(D)** Representative transmission electron microscope image of isolated Exos (scale bar = 500 nm/200 nm). **(E)** Particle size distribution by NTA. **(F)** Representative Western blotting for Exos surface marker (CD63 and TSG101). Note: ALP, alkaline phosphatase; hBMSCs, human bone marrow mesenchymal stem cells; Exos, exosome; NTA, Nanoparticle Tracking Analysis.

After three passages, the cultured cells displayed a homogeneous fibroblast-like phenotype with characteristic spindle-shaped morphology. Flow cytometric analysis confirmed that hBMSCs highly expressed the mesenchymal stem cell markers CD73 (99.93%) and CD90 (100%), while showing minimal expression of hematopoietic lineage markers CD34 (0.35%) and CD45 (0.34%), consistent with their mesenchymal identity (Figure 1).

Exos were comprehensively characterized by TEM, NTA, and Western blot. TEM images revealed the typical cup-shaped morphology of Exos, with particle diameters ranging from 40 to 150 nm (Figure 1D). NTA demonstrated a consistent particle size distribution centered around approximately 100 nm (Figure 1E). Western blot analysis confirmed the presence of canonical exosomal markers CD63 and TSG101 (Figure 1F).

### Differential expression profiles of ncRNAs, miRNAs, mRNAs in both hBMSCs and hBMSCs-Exos for SONFH and bioinformatics analysis

**FIGURE 2:**
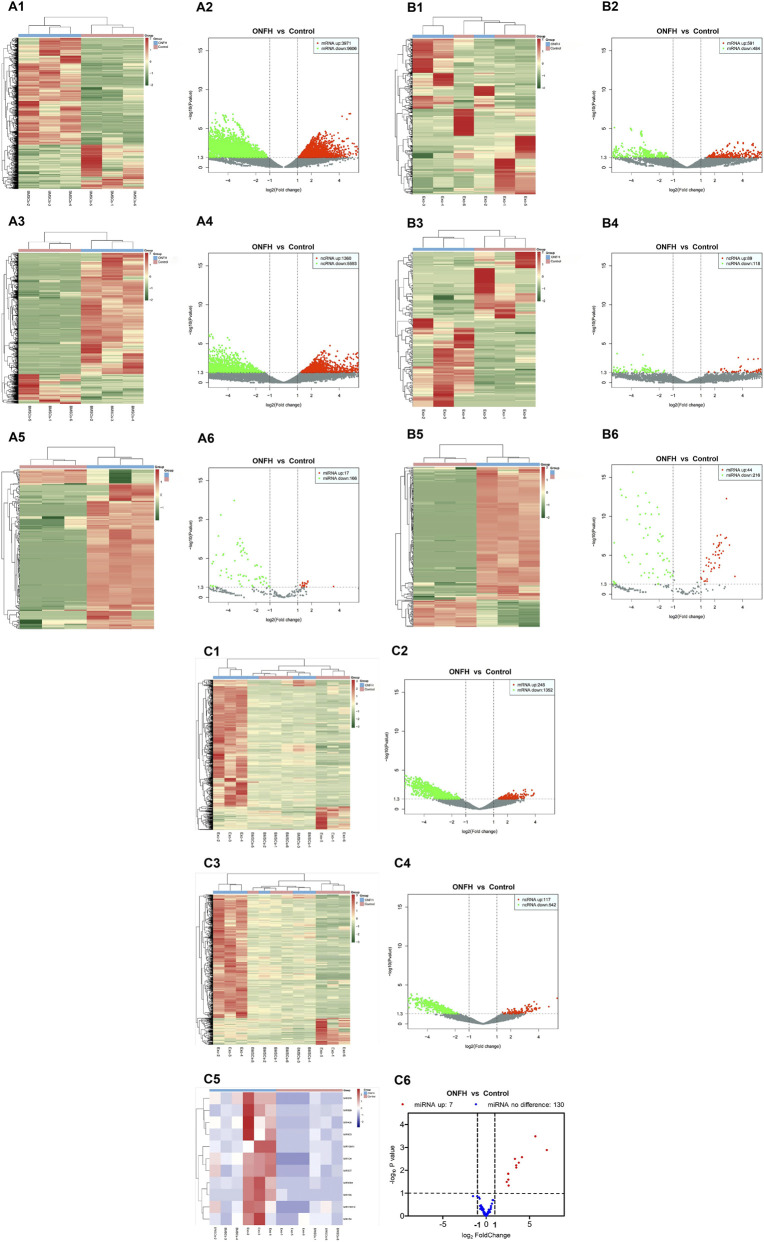
Differential expression profiles of ncRNAs, miRNAs, mRNAs in both hBMSCs and hBMSCs-Exos for SONFH. **(A1,A2)** The heat map and volcano plot showed the differentially expressed mRNAs in hBMSCs between the two groups. **(A3,A4)** The heat map and volcano plot showed the differentially expressed ncRNAs in hBMSCs between the two groups. **(A5,A6)** The heat map and volcano plot showed the differentially expressed miRNAs in hBMSCs-Exos between the two groups. **(B1,B2)** The heat map and volcano plot showed the differentially expressed mRNAs in hBMSCs-Exos between the two groups. **(B3,B4)** The heat map and volcano plot showed the differentially expressed ncRNAs in hBMSCs-Exos between the two groups. **(B5,B6)** The heat map and volcano plot showed the differentially expressed miRNAs in hBMSCs between the two groups. **(C1,C2)** The heat map and volcano plot showed the differentially expressed mRNAs in both hBMSCs and hBMSCs-Exos between the two groups. **(C3,C4)** The heat map and volcano plot showed the differentially expressed ncRNAs in both hBMSCs and hBMSCs-Exos between the two groups. **(C5,C6)** The heat map and volcano plot showed the differentially expressed miRNAs in both hBMSCs and hBMSCs-Exos between the two groups. Note: hBMSCs, human bone marrow mesenchymal stem cells; ONFH, osteonecrosis of the femoral head; Exos, exosome; ncRNAs, non-coding RNAs; miRNAs, microRNAs; mRNAs, messenger RNAs.

NGS results revealed a total of 6,953 differentially expressed ncRNAs in hBMSCs from patients with SONFH compared to controls, including 1,360 upregulated and 5,593 downregulated), 260 differentially expressed miRNAs (44 upregulated and 216 downregulated), 13,577 differentially expressed mRNAs (3,971 upregulated and 9,606 downregulated) were identified (Figure 2A1–A4, B5,B6). In hBMSCs-Exos, 207 differentially expressed ncRNAs (89 upregulated and 118 downregulated), 183 differentially expressed miRNAs (17 upregulated and 166 downregulated), and 1,075 differentially expressed mRNAs (591 upregulated and 484 downregulated) were detected (Figures 2A5,A6, B1–B4).Integrated analysis of transcripts expressed in both hBMSCs and hBMSCs-Exos identified 659 differentially expressed ncRNAs (117 upregulated and 542 downregulated), 11 differentially expressed miRNAs (all upregulated), and 1,600 differentially expressed mRNAs (248 upregulated and 1,352 downregulated) (Figures 2C1–C6).

**FIGURE 3:**
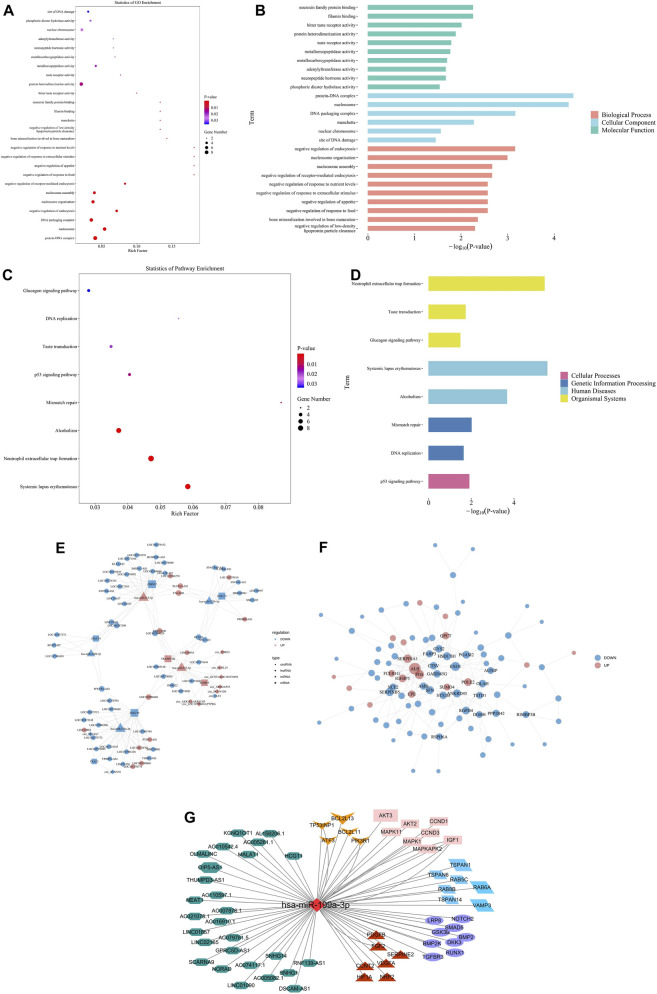
Enrichment analysis for GO and KEGG pathway, and predicted interaction network of miR-199a-3p and its target genes. **(A)** Bubble plot of GO enrichment analysis. **(B)** Bar plot showing the top enriched GO terms across BP, CC, and MF categories. **(C)** Bubble plot of KEGG pathway enrichment. **(D)** Bar plot of significantly enriched KEGG pathways grouped by functional classification. **(E)** The network of ceRNA constructed from differentially expressed circRNAs, lncRNAs, miRNAs, and mRNAs. **(F)** PPI network of differentially expressed mRNAs. **(G)** Potential target lncRNA–miRNA–mRNA centered on hsa-miR-199a-3p. In the network, green hexagons represent lncRNAs, the red diamond indicates hsa-miR-199a-3p, and various mRNAs are marked by shape and color: dark red triangles (angiogenesis), purple ellipses (osteogenesis), blue parallelograms (exosome biogenesis/secretion), pink rectangles (proliferation), and yellow concave pentagons (apoptosis). Note: GO, Gene Ontology; KEGG, Kyoto Encyclopedia of Genes and Genomes; BP, Biological Process; CC, Cellular Component; MF, Molecular Function; circRNAs, circular RNAs; lncRNAs, long non-coding RNAs; miRNAs, microRNAs. mRNAs, messenger RNAs; PPI, protein-protein interaction.

GO enrichment analysis demonstrated that these differentially expressed ncRNAs, miRNAs, and mRNAs were significantly enriched in MFs related to protein binding, receptor activity, and enzymatic functions, such as neurexin family protein binding, filamin binding, bitter taste receptor activity, taste receptor activity, neuropeptide hormone activity, and phosphoric diester hydrolase activity (*P* < 0.05). In the CC category, enrichment was observed in protein-DNA complexes, nucleosomes, DNA packaging complexes, nuclear chromosomes, and sites of DNA damage (*P* < 0.05). For BPs, significant enrichment was found in processes such as negative regulation of endocytosis, negative regulation of receptor-mediated endocytosis, negative regulation of response to extracellular stimuli, and bone mineralization involved in bone maturation, functions closely related to Exos biology and osteogenesis (Figures 3A,B).

KEGG pathway analysis highlighted enrichment in pathways including DNA replication, taste transduction, mismatch repair, and notably, the p53 signaling pathway, which is associated with cell proliferation, apoptosis, and differentiation (Figures 3C,D). In addition, the network of ceRNA was constructed from differentially expressed circRNAs, lncRNAs, miRNAs, and mRNAs (Figure 3E), and protein-protein interaction (PPI) network was constructed from differentially expressed mRNAs (Figure 3F).

### Functional analysis on predictive target lncRNAs and mRNAs of miR-199a-3p

Among the differentially expressed miRNAs, miR-199a-3p (also known as MIR199A1) was significantly upregulated in both hBMSCs and hBMSCs-Exos from patients with SONFH(log_2_ fold change = 3.47, *P* = 0.006) and was selected for further validation due to its reported involvement in cell proliferation, apoptosis, osteogenesis, and angiogenesis (Duan et al., 2011; Ren et al., 2016; Ghosh et al., 2017; Shuai et al., 2019).

Potential target long lncRNAs and mRNAs of miR-199a-3p were systematically predicted using the ENCORI database. Predicted targets were further analyzed by integrating information from the NCBI database. A total of 27 lncRNAs targeting miR-199a-3p were identified, including three lncRNAs (LINC01090, HCG11, and OIP5-AS1) previously reported to be involved in cell proliferation and apoptosis (Wang et al., 2019; Zhang et al., 2019). Additionally, 1,835 mRNAs targeting miR-199a-3p were predicted, including 3 lncRNAs (LINC01090, HCG11, and OIP5-AS1) involved in cell proliferation and apoptosis. Among these, key osteogenic regulators were identified, such as members of the TGFBR, BMP, RUNX, and SMAD families. Proliferation-associated signaling molecules comprised AKT, MAPK, and IGF; apoptosis-related factors included BCL, PIK3, and ATF; angiogenic mediators involved FGF, VEGF, and HIF; and components implicated in Exos biogenesis and secretion included members of the VAMP, TSPAN, and RAB families (Figure 3G).

### Expression validation and transfection efficiency for miR-199a-3p

**FIGURE 4:**
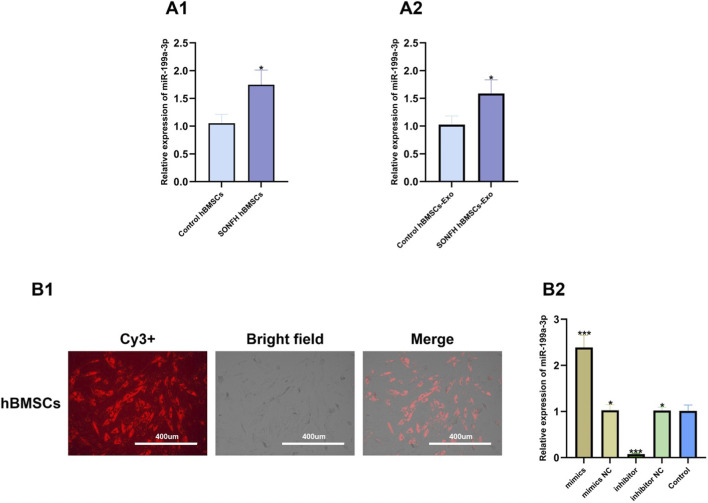
Expression level of miR-199a-3p in hBMSCs and hBMSCs-Exos from two groups and transfection efficiency for miR-199a-3p. **(A1,A2)** Relative expression level of miR-199a-3p in hBMSCs and hBMSCs-Exos from two groups. **(B1)** Images of cells 48 h after transfection, showing successful transfection (scale bar = 400 µm). **(B2)** qRT-PCR analysis of miR-199a-3p expression levels in different transfection groups. Note: hBMSCs, human bone marrow mesenchymal stem cells; SONFH, steroid-induced osteonecrosis of the femoral head; Exos, exosome; Cy3+, Cyanine 3 positive. Mimics NC, mimic negative control; Inhibitor NC, inhibitor negative control. All data were presented as the mean ± SD of three independent experiments, *p < 0.05 compared with the control group, **p < 0.01 compared with the control group, ***p < 0.001 compared with the control group, n = 9.

qRT-PCR analysis demonstrated that the expression level of miR-199a-3p in both hBMSCs and hBMSCs-Exos from patients with SONFH was significantly higher than that in control patients, consistent with the NGS results (Figure 4A). Following transfection with Cy3-labeled miR-199a-3p mimics or miR-199a-3p inhibitors, fluorescence microscopy revealed that over 90% of hBMSCs were positive for Cy3, indicating high transfection efficiency (Figure 4B1). Furthermore, qRT-PCR confirmed that miR-199a-3p expression was significantly upregulated by 130.8% in the miR-199a-3p mimics group and significantly downregulated by 92.5% in the miR-199a-3p inhibitor group compared to the non-treated, miR-NC, and miR-NCI groups, respectively (Figure 4B2).

### Suppression of miR-199a-3p promoted OB proliferation

**FIGURE 5:**
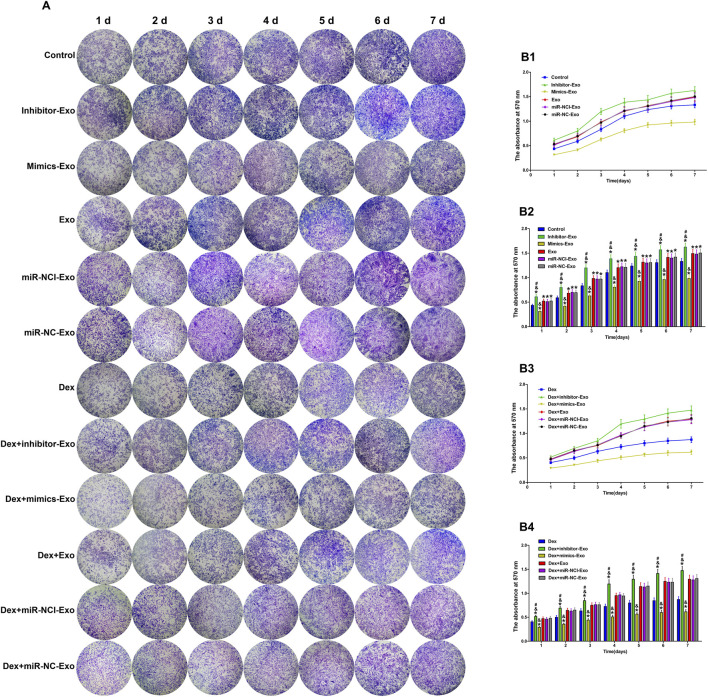
The effect of miR-199a-3p on OBs proliferation in the presence or absence of Dex (10^–6^ mol/L). **(A)** Crystal violet staining of OBs from different groups observed under a microscope. **(B1,B2)** Absorbance at 570 nm in groups treated without Dex at different time points. **(B3,B4)** Absorbance at 570 nm in groups treated with Dex at different time points. Note: d, day; Dex, dexamethasone; Exo, exosome; miR-NCI-Exo, Exos derived from cells transfected with inhibitor negative control; miR-NC-Exo, Exos derived from cells transfected with mimics negative control; OBs, osteoblasts. All data were presented as the mean ± SD of three independent experiments, *p < 0.05 compared with the control/Dex group, ^&^ *p* < 0.05 compared with miR-NCI-Exo, miR-NC-Exo, and Exo groups, ^#^ *p* < 0.05 compared with the mimics-Exo group, n = 9.

The effect of miR-199a-3p on OB proliferation was quantitatively assessed using crystal violet staining. After 7 days of continuous culture under normal conditions, cell growth was significantly increased in the miR-199a-3p inhibitor-Exo group, whereas cell proliferation was inhibited in the miR-199a-3p mimics-Exo group, compared to the control, miR-NCI-Exo, miR-NC-Exo, and Exo groups. Following continuous exposure to Dex (10^–6^ mol/L) for 7 days, consistent with previous reports (Li et al., 2021), all groups showed significantly reduced proliferation relative to the control group. However, compared to the Dex + miR-NCI-Exo, Dex + miR-NC-Exo, and Dex + Exo groups, the Dex + miR-199a-3p inhibitor-Exo group exhibited enhanced cell growth, while the Dex + miR-199a-3p mimics-Exo group showed further inhibition of proliferation (Figure 5).

These results demonstrated that the suppression of miR-199a-3p could promote OB proliferation through hBMSCs-Exos-mediated pathway and counteract the proliferation inhibition induced by high-dose Dex (10^–6^ mol/L).

### Suppression of miR-199a-3p reduced OB apoptosis

**FIGURE 6:**
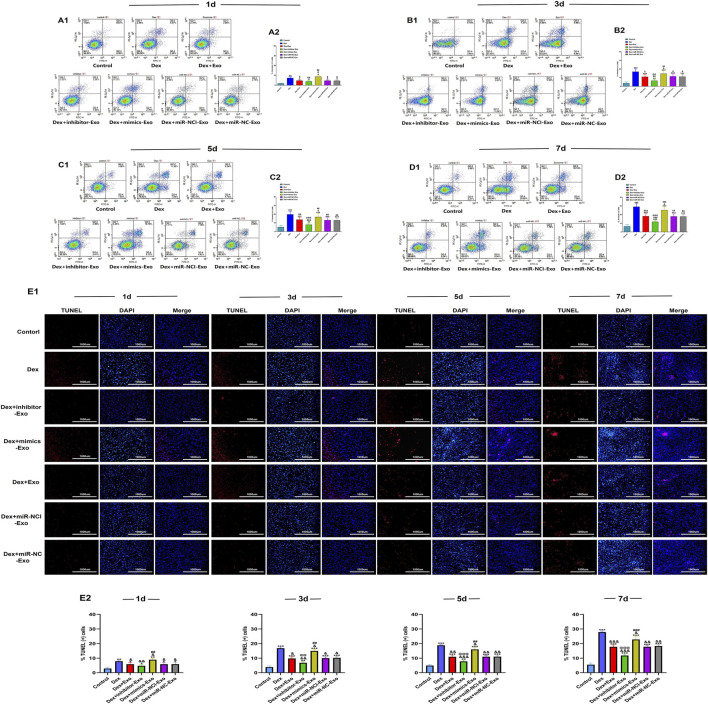
Flow cytometric analysis of apoptosis and TUNEL staining of OBs under Dex (10^–6^ mol/L). **(A1,A2)** Flow cytometric analysis and quantification of apoptosis in OBs from different groups on day 1. **(B1,B2)** Flow cytometric analysis and quantification of apoptosis in OBs from different groups on day 3. **(C1,C2)** Flow cytometric analysis and quantification of apoptosis in OBs from different groups on day 5. **(D1,D2)** Flow cytometric analysis and quantification of apoptosis in OBs from different groups on day 7. **(E)** TUNEL staining of OBs from different groups observed under a microscope and quantification of apoptotic cells (scale bar = 1,000 µm). Note: d, day; Dex, dexamethasone; Exo, exosome; miR-NC-Exo, Exos derived from cells transfected with mimics negative control; miR-NCI-Exo, Exos derived from cells transfected with inhibitor negative control; OBs, osteoblasts. All data were presented as the mean ± SD of three independent experiments, *p < 0.05 compared with the control group, **p < 0.01 compared with the control group, ***p < 0.001 compared with the control group, ^&^ *p* < 0.05 compared with the Dex group, ^&&^ *p* < 0.01 compared with the Dex group, ^&&&^ *p* < 0.001 compared with the Dex group, ^@^ *p* < 0.05 compared with the miR-NCI-Exo group, ^@@^ *p* < 0.01 compared with the miR-NCI-Exo group, ^@@@^ *p* < 0.001 compared with the miR-NCI-Exo group, ^#^ *p* < 0.05 compared with the miR-NC-Exo group, ^##^ *p* < 0.01 compared with the miR-NC-Exo group, ^###^ *p* < 0.001 compared with the miR-NC-Exo group, n = 9.

The effect of miR-199a-3p on OB apoptosis was quantitatively assessed by flow cytometric analysis and TUNEL staining. After continuous exposure to high-dose Dex (10^–6^ mol/L) for 7 days, apoptosis was significantly increased in the Dex + mimics-Exo group, whereas apoptosis was inhibited in the Dex + inhibitor-Exo group, compared to the control, Dex alone, Dex + miR-NCI-Exo, Dex + miR-NC-Exo, and Dex + Exo groups. Consistent with previous reports (Li et al., 2021), Dex exposure markedly elevated apoptosis in all treated groups compared to the control group (Figure 6).

These results demonstrated that suppression of miR-199a-3p could reduce OB apoptosis through the hBMSCs-Exos-mediated pathway and mitigate the pro-apoptotic effects induced by high-dose Dex (10^–6^ mol/L).

### Suppression of miR-199a-3p promoted osteogenesis of OBs

**FIGURE 7:**
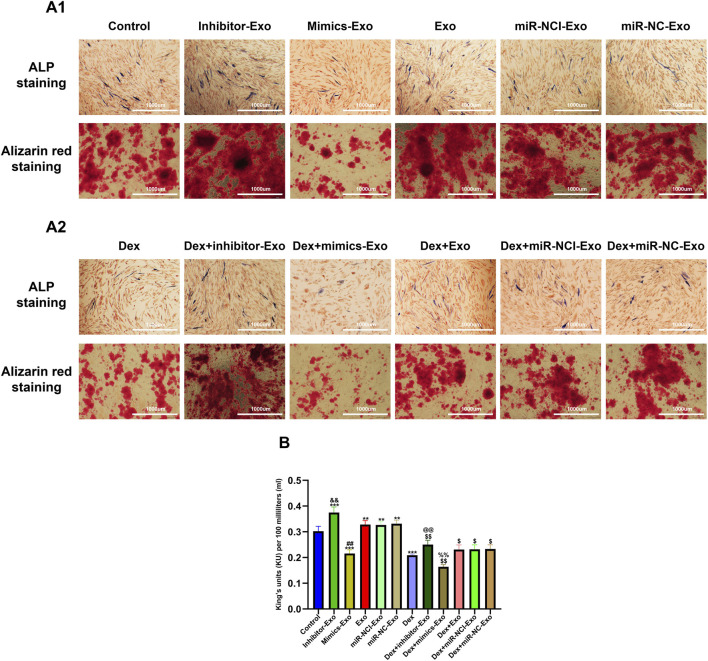
ALP staining and Alizarin red staining of OBs from different groups in the presence or absence of Dex (10^–6^ mol/L). **(A)** ALP staining and Alizarin Red staining of OBs from different groups under a microscope in the absence of Dex (10^–6^ mol/L) **(A1)** and in the presence of Dex (10^–6^ mol/L) **(A2)** (scale bar = 1,000 µm). **(B)** Quantitative analysis of ALP activity in different groups. Note: ALP, alkaline phosphatase; Dex, dexamethasone; Exo, exosome; miR-NC-Exo, Exos derived from cells transfected with mimics negative control; miR-NCI-Exo, Exos derived from cells transfected with inhibitor negative control; OBs, osteoblasts. All data were presented as the mean ± SD of three independent experiments, *p < 0.05 compared with the control group, **p < 0.01 compared with the control group, ***p < 0.001 compared with the control group, ^$^ *p* < 0.05 compared with the Dex group, ^$$^ *p* < 0.01 compared with the Dex group, ^$$$^ *p* < 0.001 compared with the Dex group, ^#^ *p* < 0.05 compared with the miR-NC-Exo group, ^##^ *p* < 0.01 compared with the miR-NC-Exo group, ^###^ *p* < 0.001 compared with the miR-NC-Exo group, ^&^ *p* < 0.05 compared with the miR-NCI-Exo group, ^&&^ *p* < 0.01 compared with the miR-NCI-Exo group, ^&&&^ *p* < 0.001 compared with the miR-NCI-Exo group, ^%^ *p* < 0.05 compared with the Dex + miR-NC-Exo group, ^%%^ *p* < 0.01 compared with the Dex + miR-NC-Exo group, ^%%%^ *p* < 0.001 compared with the Dex + miR-NC-Exo group, ^@^ *p* < 0.05 compared with the Dex + miR-NCI-Exo group, ^@@^ *p* < 0.01 compared with the Dex + miR-NCI-Exo group, ^@@@^ *p* < 0.001 compared with the Dex + miR-NCI-Exo group, n = 9.

The effect of miR-199a-3p on osteogenic differentiation was quantitatively evaluated by ALP staining, ALP activity assay, and ARS staining. Under normal culture conditions, osteogenic activity was significantly increased in the miR-199a-3p inhibitor-Exo group and decreased in the miR-199a-3p mimics-Exo group compared to the control, miR-NCI-Exo, miR-NC-Exo, and Exo groups. After continuous exposure to Dex (10^–6^ mol/L) for 14 days, consistent with previous reports (Li et al., 2021), all groups exhibited significantly reduced osteogenesis relative to the control group. However, compared to the Dex + miR-NCI-Exo, Dex + miR-NC-Exo, and Dex + Exo groups, the Dex + miR-199a-3p inhibitor-Exo group showed enhanced osteogenic differentiation, whereas the Dex + miR-199a-3p mimics-Exo group demonstrated further suppression of osteogenesis (Figure 7).

These results demonstrated that suppression of miR-199a-3p could promote osteogenic capacity in OBs through the hBMSCs-Exos-mediated pathway and counteract the inhibitory effects of high-dose Dex (10^–6^ mol/L) on osteogenesis.

### Suppression of miR-199a-3p promoted proliferation of HUVECs

**FIGURE 8:**
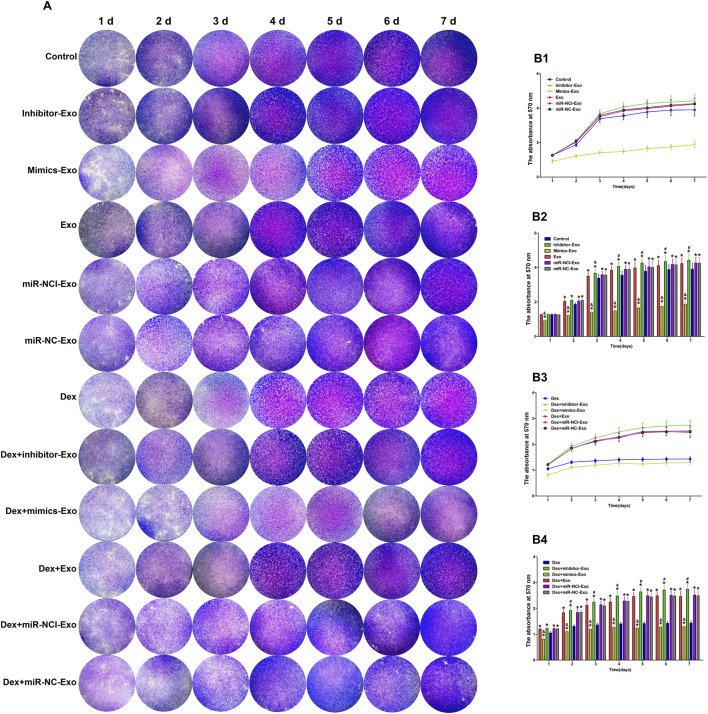
The effect of miR-199a-3p on HUVECs proliferation in the presence or absence of Dex (10^–4^ mol/L). **(A)** Crystal violet staining of HUVECs from different groups observed under a microscope. **(B1,B2)** Absorbance at 570 nm in groups treated without Dex at different time points. **(B3,B4)** Absorbance at 570 nm in groups treated with Dex at different time points. Note: d, day; Dex, dexamethasone; Exo, exosome; miR-NCI-Exo, Exos derived from cells transfected with inhibitor negative control; miR-NC-Exo, Exos derived from cells transfected with mimics negative control; HUVECs, human umbilical vein endothelial cells. All data were presented as the mean ± SD of three independent experiments, *p < 0.05 compared with the control/Dex group, ^&^ *p* < 0.05 compared with miR-NCI-Exo, miR-NC-Exo, and Exo groups, ^#^ *p* < 0.05 compared with the mimics-Exo group, n = 9.

The effect of miR-199a-3p on the proliferation of HUVECs was quantitatively assessed using crystal violet staining. After 7 days of continuous culture under normal conditions, cell proliferation was significantly increased in the miR-199a-3p inhibitor-Exo group and inhibited in the miR-199a-3p mimics-Exo group compared to the control, miR-NCI-Exo, miR-NC-Exo, and Exo groups. Similarly, under Dex (10^–4^ mol/L) treatment, the Dex + miR-199a-3p inhibitor-Exo group showed enhanced cell growth, whereas the Dex + miR-199a-3p mimics-Exo group exhibited reduced proliferation relative to the Dex + miR-NCI-Exo, Dex + miR-NC-Exo, and Dex + Exo groups (Figure 8).

These results demonstrated that suppression of miR-199a-3p could promote HUVEC proliferation through the hBMSCs-Exos-mediated pathway and mitigate the proliferation-inhibitory effects induced by high-dose Dex (10^–4^ mol/L).

### Suppression of miR-199a-3p reduced apoptosis of HUVECs

**FIGURE 9:**
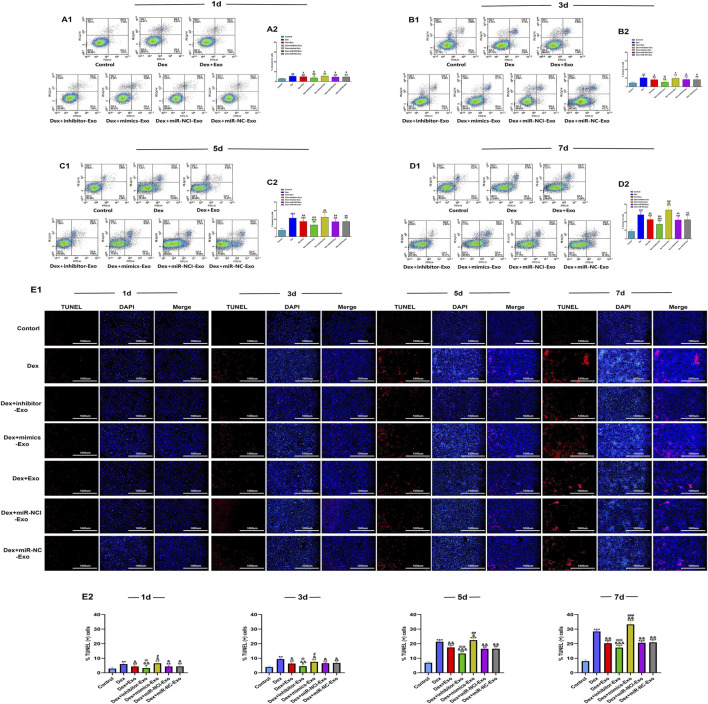
Flow cytometric analysis of apoptosis and TUNEL staining of HUVECs under Dex (10^–4^ mol/L). **(A1,A2)** Flow cytometric analysis and quantification of apoptosis in HUVECs from different groups on day 1. **(B1,B2)** Flow cytometric analysis and quantification of apoptosis in HUVECs from different groups on day 3. **(C1,C2)** Flow cytometric analysis and quantification of apoptosis in HUVECs from different groups on day 5. **(D1,D2)** Flow cytometric analysis and quantification of apoptosis in HUVECs from different groups on day 7. **(E)** TUNEL staining of HUVECs from different groups observed under a microscope and quantification of apoptotic cells (scale bar = 1,000 µm). Note: d, day; Dex, dexamethasone; Exo, exosome; miR-NC-Exo, Exos derived from cells transfected with mimics negative control; miR-NCI-Exo, Exos derived from cells transfected with inhibitor negative control; HUVECs, human umbilical vein endothelial cells. All data were presented as the mean ± SD of three independent experiments, *p < 0.05 compared with the control group, **p < 0.01 compared with the control group, ***p < 0.001 compared with the control group, ^&^ *p* < 0.05 compared with the Dex group, ^&&^ *p* < 0.01 compared with the Dex group, ^&&&^ *p* < 0.001 compared with the Dex group, ^@^ *p* < 0.05 compared with the miR-NCI-Exo group, ^@@^ *p* < 0.01 compared with the miR-NCI-Exo group, ^@@@^ *p* < 0.001 compared with the miR-NCI-Exo group, ^#^ *p* < 0.05 compared with the miR-NC-Exo group, ^##^ *p* < 0.01 compared with the miR-NC-Exo group, ^###^ *p* < 0.001 compared with the miR-NC-Exo group, n = 9.

The effect of miR-199a-3p on apoptosis of HUVECs was quantitatively assessed by flow cytometric analysis and TUNEL staining. After continuous exposure to high-dose Dex (10^–4^ mol/L) for 7 days, apoptosis was significantly increased in the Dex + mimics-Exo group and decreased in the Dex + inhibitor-Exo group compared to the control, Dex alone, Dex + miR-NCI-Exo, Dex + miR-NC-Exo, and Dex + Exo groups (Figure 9).

These results demonstrated that suppression of miR-199a-3p could reduce HUVEC apoptosis via the hBMSCs-Exos-mediated pathway and protect against Dex-induced apoptosis at high-dose Dex (10^–4^ mol/L).

### Suppression of miR-199a-3p promoted tube formation in HUVECs

**FIGURE 10:**
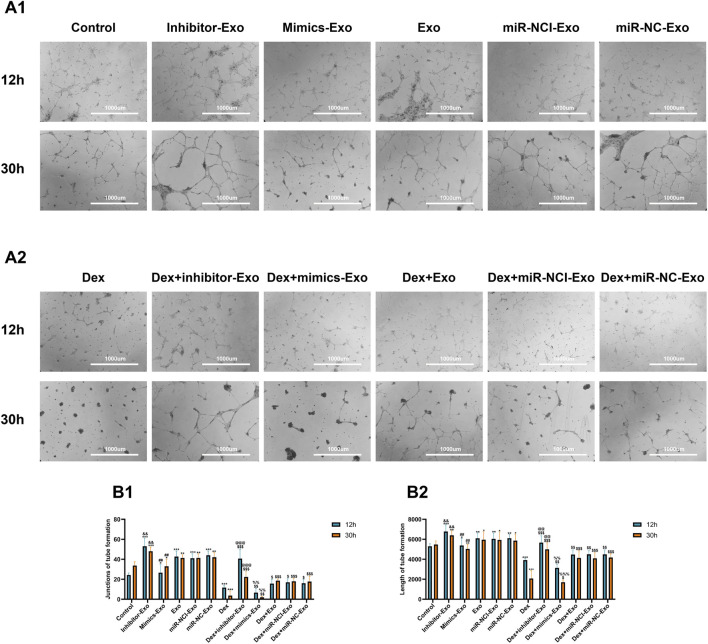
Tube formation of HUVECs from different groups in the presence or absence of Dex (10^–4^ mol/L) at 12 and 30 h. **(A)** Tube formation of HUVECs from different groups under a microscope in the absence of Dex (10^–4^ mol/L) **(A1)** and in the presence of Dex (10^–4^ mol/L) **(A2)** (scale bar = 1,000 µm). **(B)** Quantitative analysis of tube formation of HUVECs from different groups for junctions of tube formation **(B1)** and length of tube formation **(B2)**. Note: h, hour; Dex, dexamethasone; Exo, exosome; miR-NC-Exo, Exos derived from cells transfected with mimics negative control; miR-NCI-Exo, Exos derived from cells transfected with inhibitor negative control; HUVECs, human umbilical vein endothelial cells. All data were presented as the mean ± SD of three independent experiments, *p < 0.05 compared with the control group, **p < 0.01 compared with the control group, ***p < 0.001 compared with the control group, ^$^ *p* < 0.05 compared with the Dex group, ^$$^ *p* < 0.01 compared with the Dex group, ^$$$^ *p* < 0.001 compared with the Dex group, ^#^ *p* < 0.05 compared with the miR-NC-Exo group, ^##^ *p* < 0.01 compared with the miR-NC-Exo group, ^###^ *p* < 0.001 compared with the miR-NC-Exo group, ^&^ *p* < 0.05 compared with the miR-NCI-Exo group, ^&&^ *p* < 0.01 compared with the miR-NCI-Exo group, ^&&&^ *p* < 0.001 compared with the miR-NCI-Exo group, ^%^ *p* < 0.05 compared with the Dex + miR-NC-Exo group, ^%%^ *p* < 0.01 compared with the Dex + miR-NC-Exo group, ^%%%^ *p* < 0.001 compared with the Dex + miR-NC-Exo group, ^@^ *p* < 0.05 compared with the Dex + miR-NCI-Exo group, ^@@^ *p* < 0.01 compared with the Dex + miR-NCI-Exo group, ^@@@^ *p* < 0.001 compared with the Dex + miR-NCI-Exo group, n = 9.

The effect of miR-199a-3p on the tube formation ability of HUVECs was quantitatively assessed using a tube formation assay. After 30 h of continuous culture under normal conditions, increased tube formation was observed in the miR-199a-3p inhibitor-Exo group, whereas reduced tube formation was found in the miR-199a-3p mimics-Exo group compared to the control, miR-NCI-Exo, miR-NC-Exo, and Exo groups. Similarly, under Dex (10^–4^ mol/L) treatment, the Dex + miR-199a-3p inhibitor-Exo group exhibited enhanced tube formation, while the Dex + miR-199a-3p mimics-Exo group showed inhibited tube formation relative to the Dex + miR-NCI-Exo, Dex + miR-NC-Exo, and Dex + Exo groups (Figure 10).

These results demonstrated that suppression of miR-199a-3p could promote HUVEC tube formation via the hBMSCs-Exos-mediated pathway and counteract the inhibitory effects of high-dose Dex (10^–4^ mol/L) on angiogenesis.

## Discussion

Exos therapy, as a novel form of nanotherapy, holds promising advantages for hip preservation treatment in SONFH. However, the precise mechanisms by which Exos regulate target cells remain unclear. Exos, a class of extracellular vesicles and key mediators of intercellular signaling, function by carrying various types of genetic material. They transfer specific genetic information from parent cells to the extracellular space, which is then taken up by recipient cells, thereby influencing their functions and behaviors (Urbanelli et al., 2013; Qin et al., 2016; Fang et al., 2019; Masaoutis and Theocharis, 2019; Chen et al., 2021). Therefore, identifying the specific genetic cargo carried and transferred by Exos is crucial to understanding how they exert their effects via the Exos-mediated pathway.

This study identified the differential expression profiles of ncRNAs, miRNAs, and mRNAs related to the Exos-mediated pathway in SONFH by integrating differential RNAs from hBMSCs and hBMSCs-Exos obtained from patients with SONFH and controls using NGS. Our results revealed a total of 659 differentially expressed ncRNAs (117 upregulated and 542 downregulated), 11 differentially expressed miRNAs (all 11 upregulated), and 1,600 differentially expressed mRNAs (248 upregulated and 1,352 downregulated). These relatively small amounts of RNAs were expressed both in hBMSCs and hBMSCs-Exos and are considered the genetic material carried by Exos from the intracellular to the extracellular space of hBMSCs. Therefore, they are crucial for Exos to exert their regulatory effects on the biological functions and behaviors of target cells. Further bioinformatics analysis showed that these differentially expressed RNAs were involved in cell proliferation, apoptosis, osteogenesis, and Exos uptake, through pathways related to DNA damage sites, negative regulation of receptor-mediated endocytosis, response to extracellular stimuli, bone mineralization involved in bone maturation, and the p53 signaling pathway. Among the differentially expressed miRNAs, miR-199a-3p, an upregulated transcript in both hBMSCs and hBMSCs-Exos from patients with SONFH (log_2_ fold change = 3.47, *P* = 0.006), was selected for further validation due to the prediction that its downstream targets participate in cell proliferation, apoptosis, osteogenesis, angiogenesis, and Exos synthesis. miR-199a is a highly conserved microRNA across species, with its nucleotide sequence showing strong homology between humans and mice, particularly within the seed region. miR-199a-3p, one of the mature strands derived from the miR-199a precursor, contains a canonical seed sequence spanning nucleotides 2 to 8 of its mature form. Accumulating evidence indicates that miR-199a-3p plays a critical role in multiple BPs, including suppressing osteogenic differentiation, modulating osteosarcoma progression, and inhibiting angiogenesis (Ren et al., 2016; Ghosh et al., 2017; Shuai et al., 2019).

Osteogenesis and angiogenesis are well recognized as two key processes involved in both the pathogenesis and treatment of SONFH, encompassing cell proliferation, apoptosis, osteogenic and angiogenic differentiation. Previous studies have shown that long-term exposure to high doses of Dex can impair the function of BMSCs, OBs, and vascular endothelial cells (Langendorf et al., 2021), which may be one of the important pathogenic mechanism in SONFH. To mimic this, steroid-induced cell models were established using high-dose Dex treatment (10^–6^ mol/L for OBs and 10^–4^ mol/L for HUVECs) to investigate the role of miR-199a-3p-modified Exos on cell proliferation, apoptosis, osteogenesis, and angiogenesis. Our results demonstrated that suppression of miR-199a-3p could promote proliferation and inhibited apoptosis of both OBs and HUVECs via the hBMSCs-Exos-mediated pathway, while counteracting the proliferation inhibition and apoptosis induced by high-dose Dex. Additionally, suppression of miR-199a-3p enhanced osteogenesis in OBs and angiogenesis in HUVECs through the same pathway. These findings confirm the negative regulatory role of miR-199a-3p in SONFH, consistent with its elevated expression in hBMSCs and hBMSCs-Exos from patients with SONFH. The proliferation, apoptosis, and differentiation of OBs and HUVECs involve complex BPs regulated by numerous nucleic acids, proteins, and signaling pathways (Ponzetti and Rucci, 2021; Chen et al., 2022; Li et al., 2025). Predictive downstream targets of miR-199a-3p were classified into five functional categories: cell proliferation (AKT, MAPK, IGF), apoptosis (BCL, PIK3, ATF), osteogenesis (TGFBR, BMP, RUNX, SMAD), angiogenesis (FGF, VEGF, HIF), and Exos synthesis (VAMP, TSPAN, RAB). These represent potential pathways and molecular mechanisms through which miR-199a-3p may contribute to the pathogenesis of SONFH.

This study only preliminarily validated the role of miR-199a-3p-modified hBMSCs-Exo and lacks further mechanistic research. Thus, the underlying mechanisms of miR-199a-3p and its validation in SONFH animal model need further elucidation in the future research, such as the construction and validation of miR-199a-3p targeted pathway axes, the regulatory mechanism of miR-199a-3p on its downstream targets. Moreover, a small sample size was used for sequencing analysis in this study, due to limitations in obtaining clinical sample sizes. More clinical samples will be included in future studies to improve differential expression of RNAs in exosome mediated pathways in SONFH. It is worth noting that hBMSCs-Exos isolated from bone marrow was used in this sequencing analysis and amplified *in vitro* in order to reduce the interference of cell types on sequencing results, but it was not a realistic environment and the production of the Exos will be affected after culture BMSCs in a serum free media. Therefore, the future sequencing analysis will be conducted by using Exos isolated directly from the bone marrow aspirate of patients, and integrated with the sequencing results of this study to obtain a more comprehensive differential expression profile of RNAs related to SONFH. It's interesting to note that previous studies have shown that miR-199a-3p was also deregulated osteosarcoma and associated with the progression of osteosarcoma (Duan et al., 2011; Gao et al., 2015). However, the biological function of miRNAs depends on the cell type. Based on previous studies, over-expression of miR-199a-3p promotes cardiac c-kit + cell proliferation and inhibits apoptosis (Liu et al., 2017), but it also inhibits human hepatocellular carcinoma cell proliferation and promote apoptosis (Ren et al., 2016). Therefore, the tumorigenicity of miR-199a-3p needs to be paid attention to and will be further evaluated in future research.

## Conclusion

In summary, our study identified differential expression profiles of ncRNAs, miRNAs and mRNAs related to the Exos-mediated pathway in SONFH through integrated analysis. We further demonstrated the negative role of miR-199a-3p in SONFH, indicating that its suppression promotes proliferation, osteogenesis, and angiogenesis, while inhibiting apoptosis of OBs and HUVECs exposed to high-dose Dex through the hBMSCs-Exos-mediated pathway. Therefore, targeting miR-199a-3p may enhance the therapeutic efficacy of Exos-based treatments for SONFH, such as nanotherapy utilizing genetically modified Exos, screening for disease prediction biomarkers, and development of molecularly targeted drugs.

## Data Availability

The raw data supporting the conclusions of this article will be made available by the authors, upon reasonable request.

## References

[B1] Chen H. Wang L. Zeng X. Schwarz H. Nanda H. S. Peng X. (2021). Exosomes, a new star for targeted delivery. Front. Cell Dev. Biol. 9, 751079. 10.3389/fcell.2021.751079 34692704 PMC8531489

[B2] Chen W. Wu P. Yu F. Luo G. Qing L. Tang J. (2022). HIF-1alpha regulates bone homeostasis and angiogenesis, participating in the occurrence of bone metabolic diseases. Cells 11. 10.3390/cells11223552 36428981 PMC9688488

[B3] Duan Z. Choy E. Harmon D. Liu X. Susa M. Mankin H. (2011). MicroRNA-199a-3p is downregulated in human osteosarcoma and regulates cell proliferation and migration. Mol. Cancer Ther. 10, 1337–1345. 10.1158/1535-7163.MCT-11-0096 21666078 PMC3711153

[B4] Fang S. Li Y. Chen P. (2019). Osteogenic effect of bone marrow mesenchymal stem cell-derived exosomes on steroid-induced osteonecrosis of the femoral head. Drug Des. Devel Ther. 13, 45–55. 10.2147/DDDT.S178698 30587927 PMC6305133

[B5] Gado M. Baschant U. Hofbauer L. C. Henneicke H. (2022). Bad to the bone: the effects of therapeutic glucocorticoids on osteoblasts and osteocytes. Front. Endocrinol. (Lausanne) 13, 835720. 10.3389/fendo.2022.835720 35432217 PMC9008133

[B6] Gao Y. Feng Y. Shen J. K. Lin M. Choy E. Cote G. M. (2015). CD44 is a direct target of miR-199a-3p and contributes to aggressive progression in osteosarcoma. Sci. Rep. 5, 11365. 10.1038/srep11365 26079799 PMC4468826

[B7] Ghosh A. Dasgupta D. Ghosh A. Roychoudhury S. Kumar D. Gorain M. (2017). MiRNA199a-3p suppresses tumor growth, migration, invasion and angiogenesis in hepatocellular carcinoma by targeting VEGFA, VEGFR1, VEGFR2, HGF and MMP2. Cell Death Dis. 8, e2706. 10.1038/cddis.2017.123 28358369 PMC5386529

[B8] Gonda A. Kabagwira J. Senthil G. N. Wall N. R. (2019). Internalization of exosomes through receptor-mediated endocytosis. Mol. Cancer Res. 17, 337–347. 10.1158/1541-7786.MCR-18-0891 30487244

[B9] Gurunathan S. Kang M. H. Jeyaraj M. Qasim M. Kim J. H. (2019). Review of the isolation, characterization, biological function, and multifarious therapeutic approaches of exosomes. Cells 8. 10.3390/cells8040307 30987213 PMC6523673

[B10] Gurung S. Perocheau D. Touramanidou L. Baruteau J. (2021). The exosome journey: from biogenesis to uptake and intracellular signalling. Cell Commun. Signal. 19, 47. 10.1186/s12964-021-00730-1 33892745 PMC8063428

[B11] Huang C. Qing L. Xiao Y. Tang J. Wu P. (2023). Insight into steroid-induced ONFH: the molecular mechanism and function of epigenetic modification in mesenchymal stem cells. Biomolecules 14, 4. 10.3390/biom14010004 38275745 PMC10813482

[B12] Insausti C. L. Blanquer M. B. Olmo L. M. Lopez-Martinez M. C. Ruiz X. F. Lozano F. J. (2012). Isolation and characterization of mesenchymal stem cells from the fat layer on the density gradient separated bone marrow. Stem Cells Dev. 21, 260–272. 10.1089/scd.2010.0572 21504358

[B13] Kerachian M. A. Seguin C. Harvey E. J. (2009). Glucocorticoids in osteonecrosis of the femoral head: a new understanding of the mechanisms of action. J. Steroid Biochem. Mol. Biol. 114, 121–128. 10.1016/j.jsbmb.2009.02.007 19429441 PMC7126235

[B14] Kim O. Y. Lee J. Gho Y. S. (2017). Extracellular vesicle mimetics: novel alternatives to extracellular vesicle-based theranostics, drug delivery, and vaccines. Semin. Cell Dev. Biol. 67, 74–82. 10.1016/j.semcdb.2016.12.001 27916566

[B15] Langendorf E. K. Rommens P. M. Drees P. Ritz U. (2021). Dexamethasone inhibits the pro-angiogenic potential of primary human myoblasts. Int. J. Mol. Sci. 22, 7986. 10.3390/ijms22157986 34360750 PMC8348204

[B16] Lässer C. Eldh M. Lötvall J. (2012). Isolation and characterization of RNA-containing exosomes. J. Vis. Exp. 10.3791/3037 22257828 PMC3369768

[B17] Li P. Kaslan M. Lee S. H. Yao J. Gao Z. (2017). Progress in exosome isolation techniques. Theranostics 7, 789–804. 10.7150/thno.18133 28255367 PMC5327650

[B18] Li T. Xu Y. Wang Y. Jiang Y. (2021). Differential expression profiles of long noncoding RNAs and mRNAs in human bone marrow mesenchymal stem cells after exposure to a high dosage of dexamethasone. Stem Cell Res. Ther. 12, 9. 10.1186/s13287-020-02040-8 33407832 PMC7788840

[B19] Li S. Cai X. Guo J. Li X. Li W. Liu Y. (2025). Cell communication and relevant signaling pathways in osteogenesis-angiogenesis coupling. Bone Res. 13, 45. 10.1038/s41413-025-00417-0 40195313 PMC11977258

[B20] Liu J. Wang Y. Cui J. Sun M. Pu Z. Wang C. (2017). miR199a-3p regulates P53 by targeting CABLES1 in mouse cardiac c-kit(+) cells to promote proliferation and inhibit apoptosis through a negative feedback loop. Stem Cell Res. Ther. 8, 127. 10.1186/s13287-017-0515-4 28583208 PMC5460483

[B21] Livak K. J. Schmittgen T. D. (2001). Analysis of relative gene expression data using real-time quantitative PCR and the 2(-Delta Delta C(T)) method. Methods 25, 402–408. 10.1006/meth.2001.1262 11846609

[B22] Masaoutis C. Theocharis S. (2019). The role of exosomes in bone remodeling: implications for bone physiology and disease. Dis. Markers 2019, 9417914. 10.1155/2019/9417914 31485281 PMC6710799

[B23] Motta F. Timilsina S. Gershwin M. E. Selmi C. (2022). Steroid-induced osteonecrosis. J. Transl. Autoimmun. 5, 100168. 10.1016/j.jtauto.2022.100168 36213422 PMC9535426

[B24] Pant S. Hilton H. Burczynski M. E. (2012). The multifaceted exosome: biogenesis, role in normal and aberrant cellular function, and frontiers for pharmacological and biomarker opportunities. Biochem. Pharmacol. 83, 1484–1494. 10.1016/j.bcp.2011.12.037 22230477 PMC7110994

[B25] Ponzetti M. Rucci N. (2021). Osteoblast differentiation and signaling: established concepts and emerging topics. Int. J. Mol. Sci. 22, 6651. 10.3390/ijms22136651 34206294 PMC8268587

[B26] Qin Y. Wang L. Gao Z. Chen G. Zhang C. (2016). Bone marrow stromal/stem cell-derived extracellular vesicles regulate osteoblast activity and differentiation *in vitro* and promote bone regeneration *in vivo* . Sci. Rep. 6, 21961. 10.1038/srep21961 26911789 PMC4766421

[B27] Ren K. Li T. Zhang W. Ren J. Li Z. Wu G. (2016). miR-199a-3p inhibits cell proliferation and induces apoptosis by targeting YAP1, suppressing Jagged1-Notch signaling in human hepatocellular carcinoma. J. Biomed. Sci. 23, 79. 10.1186/s12929-016-0295-7 27832779 PMC5103406

[B28] Shuai Y. Yang R. Mu R. Yu Y. Rong L. Jin L. (2019). MiR-199a-3p mediates the adipogenic differentiation of bone marrow-derived mesenchymal stem cells by regulating KDM6A/WNT signaling. Life Sci. 220, 84–91. 10.1016/j.lfs.2019.01.051 30710639

[B29] Thery C. Amigorena S. Raposo G. Clayton A. (2006). Isolation and characterization of exosomes from cell culture supernatants and biological fluids. *Curr. Protoc. Cell Biol*. Chapter 3. 10.1002/0471143030.cb0322s30 18228490

[B30] Urbanelli L. Magini A. Buratta S. Brozzi A. Sagini K. Polchi A. (2013). Signaling pathways in exosomes biogenesis, secretion and fate. Genes (Basel) 4, 152–170. 10.3390/genes4020152 24705158 PMC3899971

[B31] Wang Y. Shi F. Xia Y. Zhao H. (2019). LncRNA OIP5-AS1 predicts poor prognosis and regulates cell proliferation and apoptosis in bladder cancer. J. Cell Biochem. 120, 7499–7505. 10.1002/jcb.28024 30485498

[B32] Zhang H. Huang H. Xu X. Wang H. Wang J. Yao Z. (2019). LncRNA HCG11 promotes proliferation and migration in gastric cancer *via* targeting miR-1276/CTNNB1 and activating wnt signaling pathway. Cancer Cell Int. 19, 350. 10.1186/s12935-019-1046-0 31889902 PMC6933929

